# Phenylalanine Losses in Neutralization Dialysis: Modeling and Experiment

**DOI:** 10.3390/membranes13050506

**Published:** 2023-05-11

**Authors:** Anton Kozmai, Mikhail Porozhnyy, Violetta Gil, Lasaad Dammak

**Affiliations:** 1 Membrane Institute, Kuban State University, 149, Stavropolskaya Str., 350040 Krasnodar, Russia; porozhnyj@mail.ru (M.P.); violetta_gil@mail.ru (V.G.); 2 Institut de Chimie et des Materiaux Paris-Est (ICMPE), UMR 7182 CNRS—Universite Paris-Est Creteil, 2 Rue Henri Dunant, 94320 Thiais, France; dammak@u-pec.fr

**Keywords:** ion-exchange membrane, neutralization dialysis, modeling, phenylalanine losses, amino acid, demineralization

## Abstract

A non-steady state mathematical model of an amino acid (phenylalanine (Phe)) and mineral salt (NaCl) solution separation by neutralization dialysis (ND) carried out in a batch mode is proposed. The model takes into account the characteristics of membranes (thickness, ion-exchange capacity, and conductivity) and solutions (concentration, composition). As compared to previously developed models, the new one considers the local equilibrium of Phe protolysis reactions in solutions and membranes and the transport of all the phenylalanine forms (zwitterionic, positively and negatively charged) through membranes. A series of experiments on ND demineralization of the NaCl and Phe mixed solution was carried out. In order to minimize Phe losses, the solution pH in the desalination compartment was controlled by changing the concentrations of the solutions in the acid and alkali compartments of the ND cell. The validity of the model was verified by comparison of simulated and experimental time dependencies of solution electrical conductivity and pH, as well as the concentration of Na^+^, Cl^−^ ions, and Phe species in the desalination compartment. Based on the simulation results, the role of Phe transport mechanisms in the losses of this amino acid during ND was discussed. In the experiments carried out, the demineralization rate reached 90%, accompanied by minimal Phe losses of about 16%. Modeling predicts a steep increase in Phe losses when the demineralization rate is higher than 95%. Nevertheless, simulations show that it is possible to achieve a highly demineralized solution (by 99.9%) with Phe losses amounting to 42%.

## 1. Introduction

In 1986, M. Igawa et al. proposed [1] a new desalination method based on the principle of Donnan dialysis. In a three-compartment cell, the electrolyte solution was separated from the HCl solution using a cation-exchange membrane (CEM) and from the NaOH solution using an anion-exchange membrane (AEM). Cations and anions were exchanged with protons and hydroxide ions through the corresponding membranes, simultaneously providing the processes of desalination and neutralization. Later, the same authors suggested calling this method neutralization dialysis (ND) and demonstrated its applicability for the mutual separation of electrolytes and non-electrolytes in the example of the mixed aqueous solution of potassium chloride and methyl alcohol [2].

The ND method does not require applying an electric field (such as electromembrane methods) and high pressure (such as baromembrane methods). In general, ND is characterized by low energy costs and ease of technical implementation and does not demand high investments, which determines its prospects in terms of application in diverse fields. To date, ND has been found to be used in a wide range of different applications, for example, to neutralize acidified effluents from enterprises in various industries [3], purification of tap water [4], and surface water desalination for producing potable water [5]. In recent years, ND has been introduced as a basic element of the energy-harvesting technique utilizing waste acid and alkaline solutions (capacitive neutralization dialysis) [6]. Of particular interest is the use of ND for the separation of multicomponent mixtures and the separation of pure valuable components. This method has shown its effectiveness for the separation of weak acids and bases [7], isolation of mono-, oligo- and polysaccharides [8], aldehydes [9], and amino acids [8,10,11]. Another prospect for ND may be its application for the separation of organic and mineral compounds as a pretreatment step for electrodialysis processing of liquids of biological origin, for example, whey. This will allow solving the problem of depressed ion transport due to the presence of organic substances [12,13].

In terms of purification and mutual separation of amino acids, ND has certain advantages over other methods. Amino acids are ampholytes, that is, they have both acidic and basic groups in their structure and can enter the proton-transfer reactions. Such substances are characterized by different values of the equilibrium constants for protonation/deprotonation reactions. The relation of these constants determines the pH values of the medium at which amino acids can change their form (zwitterionic, positively charged, negatively charged). The main advantage of ND is the ability to control the pH value of the processed solution without adding reagents. This allows controlling the form of the ampholytes and, consequently, their fluxes across the membranes. Thus, using ND, it is possible to carry out effective demineralization, concentration, and fractionation of amino acids in the food, pharmaceutical, and biochemical industries, where the introduction of additional reagents can adversely affect the quality of the final product.

However, maintaining the required pH value of the solution being desalted is a demanding task. This value is influenced by a large number of parameters of ion-exchange membranes (for example, the ion-exchange capacity, nature of functional groups, thickness, degree of matrix crosslinking) and of the ND process (concentration and flow rate of acid, alkali solutions, and solution being desalinated in the corresponding compartments) [5,10,11,14,15]. In contrast to ampholyte-containing solutions, the mechanisms of strong electrolyte ions transfer during the ND have been studied sufficiently to gain a comprehensive idea of what trends the pH of the solution being desalted obeys. In addition to a fairly large number of experimental works, a number of mathematical models have been proposed to explain the effects arising in such systems [14,16,17]. It is noted in [5,14] that the pH of a being desalted solution of a strong binary electrolyte can change from acidic to alkaline values (or vice versa, from alkaline to acidic). This occurs due to a change in the kinetics of ion exchange from internal diffusion (transfer is limited by the membrane) to external diffusion (transfer is limited by the diffusion boundary layers), which determines the rate of exchange across the ion-exchange membranes. The study [17] showed that strong pH variations occur back and forth multiple times and are associated with changes in the rate of exchange across the CEM and AEM caused by changes in the concentration of the solution being desalinated. Thus, understanding the mechanisms of the ND process allows for controlling the exchange rate across the ion-exchange membranes and managing the pH values by varying the conditions of the process.

Knowledge about the trends of pH behavior in ND of ampholyte-containing solutions is fragmentary. It is known that pH behaves differently compared to the case of strong electrolytes, which is due to the buffer capacity of ampholytes and their ability to enter into protonation/deprotonation reactions [11,18]. The transport mechanisms in such systems are more complex due to the interaction of a large number of particles, the conjunction of their fluxes, and changes in the form of ampholyte species. Thus, pH control in the case of ampholyte-containing solutions is a difficult task. The selection of optimal conditions for the ND process to minimize the loss of target components through an exclusively experimental approach is rather time, labor, and resource-consuming. For these purposes, the use of mathematical modeling is of great importance. The modeling will assist in deepening the understanding of the transport mechanisms and help to predict the effect of changing one or another process parameter.

An attempt to simulate the process of demineralization of an ampholyte-containing solution (a mixture of phenylalanine and mineral salt) during ND was made in [18]. The model assumes that ampholyte species are not transferred across the membranes. They only can enter into protonation/deprotonation reactions in the desalination compartment due to H^+^ and OH^−^ ions transfer through the CEM and AEM, respectively. This model did not allow for estimating the losses of the ampholyte during solution demineralization, as well as describing the mechanisms of ampholyte species transport across the membranes.

In our previous work [11], we proposed a mathematical model describing the process of neutralization dialysis of the mixed solution of phenylalanine (Phe) and sodium chloride. The model considered local changes in the concentration of mineral salt ions and Phe species (in the form of cation, anion, and zwitterion) in the desalination compartment. Additionally, the transport of charged forms of Phe across the membranes and diffusion boundary layers (DBLs) was taken into account. Theoretically found values of amino acid losses were lower than the experimental ones. This difference can be explained by the fact that the model does not take into account the transfer of the zwitterionic form of Phe across membranes. The membrane’s internal pH differs by 1–2 units from that of the external solution [19]. When entering the membrane, the charged forms of Phe appear, implementing the facilitated transport mechanism [20,21].

The main goal of this work is to assess the applicability of ND to the demineralization of a mixed mineral salt and amino acid solution in terms of amino acid losses. For this purpose, we propose a new non-steady state mathematical model to describe the Phe and NaCl mixture demineralization by the ND method in the batch mode. Unlike the previous model [11], the new one takes into account the transfer of not only charged forms but also the zwitterionic form of Phe across the membranes and DBLs. The ability of amino acid to change its electric charge due to protonation/deprotonation reactions and the local changes in the concentration of Na^+^, Cl^−^, H^+^, OH^−^ ions, and Phe species (in charged and zwitterionic forms) are also considered. The developed model takes into account the main characteristics of membranes (thickness, ion-exchange capacity, and electrical conductivity) and solutions (concentrations, diffusion coefficients of components, and equilibrium constants for protonation/deprotonation reactions), as well as the flow rate of the solutions in the dialyzer compartments. We show that the model adequately describes the experimental data obtained for the equimolar mixture NaCl + Phe in the series of experiments where the initial acid and alkali concentrations were varied. We also demonstrate the ability of the new model to predict the ND process behavior and amino acid losses if experimental data are limited.

## 2. Theoretical

### 2.1. System under Study

**Figure 1:**
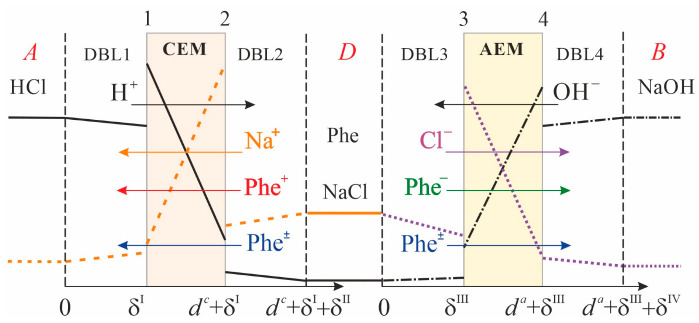
Schematic representation of the modeled system geometry. DBL1 and DBL2 are the diffusion boundary layers adjacent to the CEM from the side of the acid and desalination compartments, respectively; DBL3 and DBL4 are diffusion boundary layers adjacent to the AEM from the side of the desalination and alkali compartments, respectively; indices 1, 2, 3, and 4 denote the boundaries of the CEM and AEM marches with the corresponding compartments.

The system under study consists of three compartments: acid (*A*), alkali (*B*), and desalination compartment (*D*), separated from each other by the CEM and AEM (Figure 1).

Each of the circuits formed by the corresponding compartments together with the elements of the hydraulic system (tubes, intermediate tanks) has volumes *V^A^*, *V^B^*, and *V^D^* for the acid, alkali, and desalination compartments, respectively. Solutions of acid (HCl), alkali (NaOH), and mixed solution (NaCl + Phe) circulate through compartments *A*, *B,* and *D*, respectively, and through intermediate tanks.

Convective transport within the DBLs is neglected. Implicitly, it is taken into account by setting the DBL thickness. It is assumed that all the DBLs have equal thickness. This is justified by the same hydrodynamic conditions in the compartments. It is assumed that the concentrations of all ions do not change along the desalination compartment. In other words, the concentrations of ions at any moment in time are the same both in the volumes of the compartments and in the corresponding intermediate tanks. In the conditions of the experiment described in Section 3, these assumptions are justified by the short length of the cell (2.7 cm) and the pipes (60 cm) used in the hydraulic circuit, as well as a relatively high velocity of solution flowing. The average time of solution passage through the *D* compartment (about 2 s) as well as through the overall circuit (about 40 s) is small compared to the duration of a single experimental run (86,000 s). During 40 s, the concentration of the solution in the circuit may change only by 0.02%, which is small compared to the concentration measurement error. Therefore, the material balance equations are applied to the whole volume of solutions circulating through acid, alkali, and desalination circuits.

In contrast to the simplification used in work [18], the concentration of phenylalanine changes in the course of ND in membranes, desalination, acid, and alkali compartments. In contrast to the simplification used in work [11], the local equilibrium of Phe protonation/deprotonation reactions in membranes and solutions, as well as the transport of its charged and zwitterionic forms in membranes and DBLs, are taken into account.

At the membrane/solution interfaces, a local thermodynamic equilibrium between counterions is assumed. CEM and AEM are assumed to be ideally selective (the transport of co-ions is neglected), which is due to the use of relatively dilute solutions in the study.

### 2.2. Problem Formulation

The following set of equations describes one-dimensional ion electrodiffusion transport in the membranes and the DBLs:

the Nernst-Planck equation

(1) $$J_{j}=-D_{j}\left(\frac{\partial C_{j}}{\partial x}+z_{j}C_{j}\frac{F}{RT}\frac{\partial \varphi }{\partial x}\right)$$

the electroneutrality condition

(2) $$\underset{j}{\Sigma }z_{j}C_{j}=\omega Q$$

the condition of zero current flow

(3) $$\underset{j}{\Sigma }z_{j}J_{j}=0$$

the equation of material balance

(4) $$\frac{\partial C_{j}}{\partial t}=-\frac{\partial J_{j}}{\partial x}$$

where $C_{j}$ is the concentration, $J_{j}$ is the flux density, $z_{j}$ is the charge, $D_{j}$ is the diffusion coefficient of ion j; Q is the membrane ion-exchange capacity, ω can take the values −1, +1, or 0 for an AEM, a CEM, and a solution, respectively; t is the time, x is the coordinate normal to the membranes surfaces, R, T, and F are the gas constant, temperature, and Faraday constant, respectively. In the *D* compartment j = H^+^, OH^−^, Na^+^, Cl^−^, Phe^+^, Phe^−^, Phe^±^; in the *A* compartment and in CEM j = H^+^, Na^+^, Phe^+^, Phe^±^; in the *B* compartment and in AEM j = OH^−^, Cl^−^, Phe^−^, Phe^±^.

Hereinafter, the zwitterion (N+H3−CH(CH2C6H5)−COO−), the cation (N+H3−CH(CH2C6H5)−COOH), and the anion ($NH_{2}-CH\left(CH_{2}C_{6}H_{5}\right)-COO^{-}$) of Phe are denoted as Phe^±^, Phe^+^, and Phe^−^, respectively.

The equilibrium between H^+^, OH^−^ ions, and water molecules in solution is described by the equation:

(5) $$K_{w}=C_{H}^{}\cdot C_{OH}^{}=10^{-14}\;{\mathrm{mol}}^{2}\cdot L^{-2}$$

Phenylalanine enters into the protolysis reactions:

(6) $$Phe^{+}+H_{2}O↔Phe^{\pm }+H_{3}O^{+}$$

(7) $$Phe^{\pm }+H_{2}O↔Phe^{-}+H_{3}O^{+}$$

The chemical equilibrium constants of reactions (6), $K_{1}$, and (7), $K_{2}$, at 25 °C are expressed as follows:

(8) $$K_{1}=\frac{\left[Phe^{\pm }\right]\left[H_{3}O^{+}\right]}{\left[Phe^{+}\right]}=6.31\cdot 10^{-3}\;\mathrm{mol}\cdot L^{-1}$$

and

(9) $$K_{2}=\frac{\left[Phe^{-}\right]\left[H_{3}O^{+}\right]}{\left[Phe^{\pm }\right]}=4.90\cdot 10^{-10}\;\mathrm{mol}\cdot L^{-1}$$

respectively [22].

**Figure 2:**
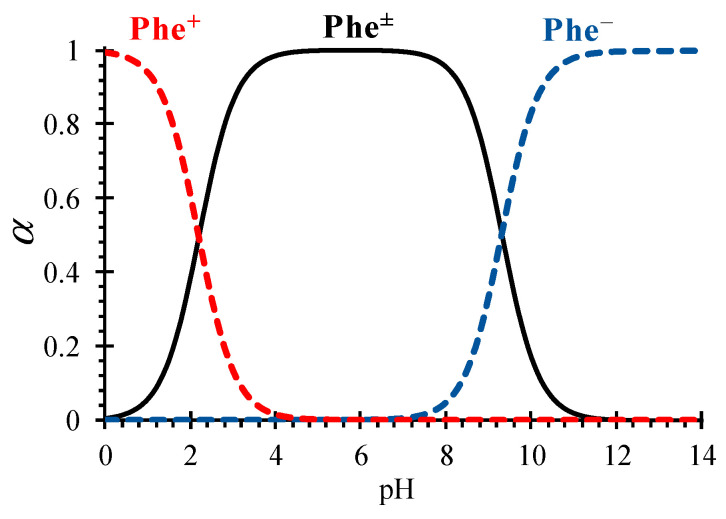
Mole fractions (α) of Phe species in an aqueous solution as function of pH, calculated from Equations (10)–(12).

The local change in the concentration of Phe species (Phe^±^, Phe^+^, and Phe^−^) are calculated by Equations (8) and (9) as follows:

(10) $$C_{Phe^{+}}=\frac{C_{Phe^{tot}}}{\frac{K_{1}K_{2}}{C_{H}^{2}}+\frac{K_{1}}{C_{H}}+1}$$

(11) $$C_{Phe^{-}}=\frac{K_{1}K_{2}C_{Phe^{+}}}{C_{H}^{2}}$$

(12) $$C_{Phe^{\pm }}=\frac{K_{1}C_{Phe^{+}}}{C_{H}}$$

where $C_{Phe^{tot}}=C_{Phe^{\pm }}+C_{Phe^{+}}+C_{Phe^{-}}$ is the total concentration of all Phe species [18,20]. Thus, depending on the local pH value of the solution in the ND system, Phe changes its form due to protolysis reactions (Figure 2).

Changes in ion concentrations in compartments *A*, *D*, and *B*, as well as the local ion concentrations in DBLs and membranes, are calculated from the system of equations formed by the material balance condition, Equation (4), taking into account Equations (6) and (7):

(13) $$\frac{\partial C_{H}}{\partial t}=-divJ_{H}+k_{1}C_{Phe^{+}}-k_{-1}C_{Phe^{\pm }}C_{H}+k_{2}C_{Phe^{\pm }}-k_{-2}C_{Phe^{-}}C_{H}+k_{d}C_{H_{2}O}-k_{r}C_{H}C_{OH}$$

(14) $$\frac{\partial C_{OH}}{\partial t}=-divJ_{OH}+k_{d}C_{H_{2}O}-k_{r}C_{H}C_{OH}$$

(15) $$\frac{\partial C_{Phe^{+}}}{\partial t}=-divJ_{Phe^{+}}-k_{1}C_{Phe^{+}}+k_{-1}C_{Phe^{\pm }}C_{H}$$

(16) $$\frac{\partial C_{Phe^{-}}}{\partial t}=-divJ_{Phe^{-}}+k_{2}C_{Phe^{\pm }}-k_{-2}C_{Phe^{-}}C_{H}$$

(17) $$\frac{\partial C_{Phe^{\pm }}}{\partial t}=-divJ_{Phe^{\pm }}+k_{1}C_{Phe^{+}}-k_{-1}C_{Phe^{\pm }}C_{H}-k_{2}C_{Phe^{\pm }}+k_{-2}C_{Phe^{-}}C_{H}$$

(18) $$\frac{\partial C_{Na}}{\partial t}=-divJ_{Na}$$

(19) $$\frac{\partial C_{Cl}}{\partial t}=-divJ_{Cl}$$

where *k*_1_, *k*_2_ are the dissociation rate constants, *k*_−1_, *k*_−2_ are the recombination rate constants in reactions described by Equations (6) and (7), respectively; *k_d_*, *k_r_* are the dissociation and recombination rate constants of water.

Solving the system of Equations (1)–(3) and (13)–(19) allows one to find the change in concentrations of H^+^, Na^+^, Phe^+^, Phe^±^ ions in the acid compartment, CEM, and adjacent DBLs (DBL1 and DBL2); OH^−^, Cl^−^, Phe^−^, Phe^±^ ions in the alkali compartment, AEM, and adjacent DBLs (DBL3 and DBL4); H^+^, OH^−^, Na^+^, Cl^−^, Phe^+^, Phe^−^, Phe^±^ ions in the desalination compartment.

The boundary conditions imply the ion exchange equilibrium and the flux continuity condition at the membrane/solution interfaces.

Local equilibrium at the membrane/solution interfaces is described by the equations:

(20) $$K_{H,Na}^{c}=C_{H}^{c}C_{Na}/\left(C_{Na}^{c}C_{H}\right)$$

(21) $$K_{H,Phe^{+}}^{c}=C_{H}^{c}C_{Phe^{+}}/\left(C_{Phe^{+}}^{c}C_{H}\right)$$

(22) $$K_{OH,Cl}^{a}=C_{OH}^{a}C_{Cl}/\left(C_{Cl}^{a}C_{OH}\right)$$

(23) $$K_{OH,Phe^{-}}^{a}=C_{OH}^{a}C_{Phe^{-}}/\left(C_{Phe^{-}}^{a}C_{OH}\right)$$

where $C_{j}^{c}$ and $C_{j}^{a}$ are the concentration of ion j at the membrane solution interface from the inside of the membrane (superscripts “*c*” and “*a*” relates to CEM and AEM, respectively); $K^{c}$ is the ion exchange equilibrium constant for H^+^/Na^+^ and H^+^/Phe^+^ (at the CEM surface from the inside of the membrane); $K^{a}$ is the similar parameter for OH^−^/Cl^−^ and OH^−^/Phe^−^ (at the AEM surface from the inside of the membrane).

The continuity of the flux condition at the CEM/solution boundaries reads as:

(24) $${J_{j}|}_{x=\delta^{I}}={J_{j}^{c}|}_{x=\delta^{I}},\;{J_{j}|}_{x=d^{c}+\delta^{I}+\delta^{II}}={J_{j}^{c}|}_{x=d^{c}+\delta^{I}+\delta^{II}}$$

At the DBLs/solutions boundaries, the concentration continuity condition is set:

(25) $${\left(C_{j}\right)}_{x=0}=C_{j}^{A},\;{\left(C_{j}\right)}_{x=d^{c}+\delta^{I}+\delta^{II}}=C_{j}^{D}$$

where $C_{j}^{A}$, $C_{j}^{D}$ are the concentration of ion j in *A* and *D* compartments, respectively.

Similar boundary conditions are set from the AEM side.

The system of the partial differential equations described above was solved numerically using Matlab software.

At the beginning of the ND process (at *t* = 0), a uniform distribution of concentrations in the diffusion layers is assumed, equal to the initial concentrations of the feed solutions. Generally, the initial conditions read as

(26) $${C_{j}\left(x\right)|}_{t=0}=\left\{ \begin{array}{l} C_{j}^{0},\;0\leq x\leq \delta^{I}\;\mathrm{for}\;\mathrm{the}\;\mathrm{DBL}1\\ C_{j}^{0},\;d^{c}+\delta^{I}\leq x\leq d^{c}+\delta^{I}+\delta^{II}\;\mathrm{for}\;\mathrm{the}\;\mathrm{DBL}2\\ C_{j}^{0},\;0\leq x\leq \delta^{III}\;\mathrm{for}\;\mathrm{the}\;\mathrm{DBL}3\\ C_{j}^{0},\;d^{a}+\delta^{III}\leq x\leq d^{a}+\delta^{III}+\delta^{IV}\;\mathrm{for}\;\mathrm{the}\;\mathrm{DBL}4\\ C_{j}^{c}\left(x\right),\;\delta^{I}\leq x\leq d^{c}+\delta^{I}\;\mathrm{for}\;\mathrm{the}\;\mathrm{CEM}\\ C_{j}^{a}\left(x\right),\;\delta^{III}\leq x\leq d^{a}+\delta^{III}\;\mathrm{for}\;\mathrm{the}\;\mathrm{AEM} \end{array}\right.$$

where $C_{j}^{0}$ is the initial concentration of species j in the corresponding compartment,$C_{j}^{c}\left(x\right)$ and $C_{j}^{a}\left(x\right)$ are linear functions that distribute the concentration of species j linearly between left-hand and right-hand membrane boundaries.

### 2.3. Parameters of the Model

The input parameters of the model can be conventionally divided into three groups: thermodynamic, kinetic, and those that characterize the nature of the ion-exchange material. There are also several input parameters characterizing the solution and the ND system: electrolyte concentrations, pH, and DBLs thickness.

Thermodynamic parameters include ion exchange equilibrium constants between membranes and external solutions and chemical equilibrium constants of Phe protonation/deprotonation reactions. For simplicity, the ion exchange equilibrium constants are taken equal to 1.

Kinetic parameters include the diffusion coefficients of mineral salt and Phe species in membranes and solutions.

The parameter characterizing the nature of the membrane material is the membrane ion-exchange capacity.

The output parameters of the model are the concentrations of ions and Phe species in the membranes, DBLs, and in compartments of the studied system.

The input parameters of the system under study were obtained from independent experiments or taken from the literature. The values of the fitting parameters were found from the condition of the best fit between the simulated and experimental time dependencies of pH, electrical conductivity, and concentrations of mineral salt ions and Phe in the solution in the *D* compartment. The fitting parameters of the model are the diffusion coefficients of ions and Phe species in membranes (${\bar{D}}_{j}$) and the thickness of DBLs (δ).

Note that the order of magnitude of ${\bar{D}}_{j}$ is estimated from experimental data on the membrane electrical conductivity using the Nernst-Einstein relation, as seen in work [17].

## 3. Experimental

### 3.1. Neutralization Dialysis Process

Neutralization dialysis was carried out in a three-compartment dialysis cell (similar to one used in [11]) consisting of acid and alkali compartments, as well as a desalination compartment separated by a CEM and an AEM. In each of the compartments, a spacer of the width equal to the intermembrane distance (0.6 cm) was placed. The membrane’s working surface area was 7.29 cm^2^ (2.7 cm × 2.7 cm).

The average linear flow rate of the solutions in each compartment was 1.5 cm·s^−1^. A total of 2 L of HCl solution and 2 L of NaOH solution of various concentrations, respectively, circulated through the acid and alkali compartments. A total of 0.5 L of a mixed solution of NaCl and Phe containing dissolved components in an equimolar ratio (0.02 mol·L^−1^ NaCl + 0.02 mol·L^−1^ Phe) was circulated through the desalination compartment.

The initial pH value of the NaCl + Phe mixed solution was 5.5–5.6, which is close to the Phe isoelectric point (pI = 5.76). Such conditions (according to the estimates by Equations (10)–(12)) provide the molar fraction of the zwitterionic form of Phe in the mixed solution in the range from 99.93% to 99.94%.

In the course of neutralization dialysis, the pH and electrical conductivity of the solution being demineralized were measured using an Expert-001 pH meter and an Expert-002 conductometer (JSC Econiks-Expert, Moscow, Russia). The experiments were carried out at a temperature of 25.0 ± 0.5 °C.

To assess the loss of Phe and the demineralization rate during ND, sampling of the solution in the *D* compartment was carried out after 12 and 24 h (in the middle and at the end of the experiment). The Phe concentration was determined using the UV-1800 spectrophotometer (TM ECO-VIEW, Shanghai Mapada Instruments Co., Ltd., Shanghai, China) at a wavelength of 259 nm. The content of Na^+^ and Cl^−^ ions was determined using a DIONEX ICS-3000 (Thermo Fisher Scientific, Waltham, MA, USA) chromatographic system.

### 3.2. Membranes

New commercial homogeneous membranes manufactured by ASTOM (Tokyo, Japan) were used in the ND process: a cation-exchange membrane CSE and an anion-exchange membrane ASE. These are standard-grade, homogeneous membranes with high mechanical strength. The polymer matrix of the CSE membrane is a styrene-based copolymer with −SO^3−^ functional groups; the ASE membrane containing strongly basic functional groups is produced from a copolymer of styrene and divinylbenzene. In the case of both membranes, the reinforcing net is made of a mixture of polyethylene and polypropylene [23].

Some physicochemical characteristics of the CSE and ASE membranes are shown in Table 1.

## 4. Results and Discussions

The behavior of the ND system under conditions similar to those applied in the present research (concentrations of the acid and alkali solutions, average linear velocity of the solutions in the compartments, design of the dialyzer) is well studied for the case of a strong binary 1:1 electrolyte [5,14,17].

The peculiarity of the ND system is possible fluctuations of the solution pH in the *D* compartment. These fluctuations may appear as a result of (1) a change in the kinetics of ion transfer from internal to external diffusion (the ion flux is limited by transport through membranes and through DBLs, respectively) with a dilution of the solution in the *D* compartment [14] and (2) a delay in the formation of concentration profiles in membranes and DBLs as a response to changes in the concentration of the solution in the *D* compartment [17].

If an ampholyte (for example, phenylalanine) is present in the *D* compartment, the pH fluctuations become less pronounced due to the buffering effect of Phe (which enters the protonation/deprotonation reactions with H^+^ and OH^−^ ions) [18]. In this case, charged species of Phe (Phe^+^ and Phe^−^) are formed in the solution and membranes. Phe^+^ and Phe^−^ are transported through the corresponding membranes as counterions, along with the diffusion of the zwitterionic form (Phe^±^), leading to the loss of this amino acid. Therefore, pH control of the solution being desalinated is an important task in the implementation of ND of ampholyte-containing solutions.

In order to reduce the loss of Phe, it is necessary to ensure the reagent-free pH control in the *D* compartment so that the pH value is as close as possible to the pI of the amino acid. Based on the literature [8,10,17,18], pH can be controlled by influencing the ion exchange rate (ion fluxes) across the CEM and AEM. This may be realized in several ways:

(1) by the selection of membranes with desired properties (structure, thickness, ion-exchange capacity, nature of fixed ion-exchange groups, etc.);
(2) by changing the hydrodynamic conditions in the compartments of the ND system, which affects the DBL thickness near the membrane surfaces;
(3) by changing the concentration of acid and/or alkali in the corresponding compartments, which affects the concentration gradient between these compartments and the *D* compartment and, as a consequence, the ion fluxes.

In the authors’ opinion, the latter way is the simplest from a practical point of view and easiest in terms of implementation. In the present research, in order to verify the proposed model, a series of experiments with the varied concentration of acid and/or alkali in different runs were conducted.

### 4.1. pH Fluctuations in Desalination Compartment

In general, the pH behavior depends on the exchange rate (ER) ratio across the CEM + adjacent DBLs and AEM + adjacent DBLs [14,17]. The ER across the AEM + DBLs may be significantly higher or significantly lower than that across the CEM (for example, due to the great difference in concentrations in compartments *A* and *B*, membrane thickness, ion-exchange capacity, etc.), leading, respectively, to the situation where the pH in the *D* compartment will have high or low values [14].

The conditions of the ND experiment set in this work refer to the case of comparable values of the ER across the CEM and AEM. Three cases of acid and alkali solution concentrations are considered (Table 2).

**Figure 3:**
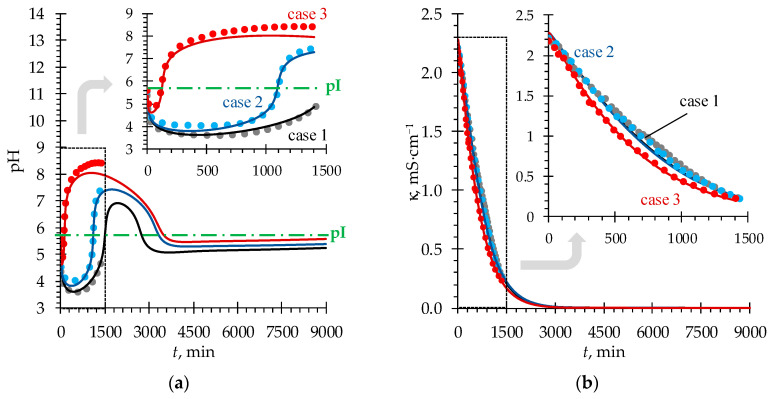
Experimental (dots) and simulated (solid lines) time dependencies of pH (**a**) and electrical conductivity (**b**) of the solution in the *D* compartment.

As can be seen from Figure 3, the experimental and simulated (using the developed model) time dependencies of pH and electrical conductivity in the *D* compartment are in good agreement in the time range where the experimental data is available. At the same time, Figure 3 shows further pH and electrical conductivity behavior predicted using the developed model. The values of model parameters used in simulations are listed in Table A1, Appendix A.

**Figure 4:**
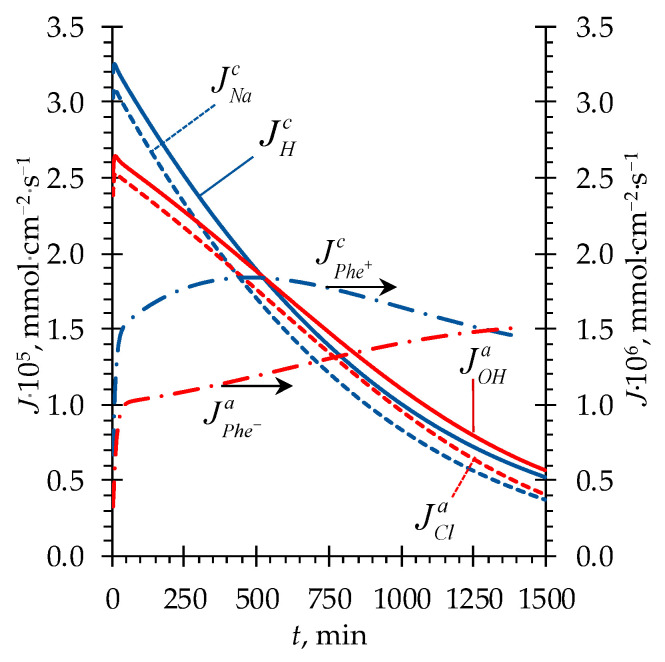
Simulated time dependencies of ion fluxes through the CEM ($J_{j}^{c}$, j = H^+^, Na^+^, Phe^+^) and AEM ($J_{j}^{a}$, j = OH^−^, Cl^−^, Phe^−^). Case 1 is considered (Table 2).

Figure 3a shows that regardless of the initial values of $C_{HCl}^{A}$ and $C_{NaOH}^{B}$ used in our study, the solution pH in the *D* compartment decreases at the beginning of the process (in all three cases). At a sufficiently high concentration of the solution being desalted at the beginning of the ND process, the limiting stage is ion transfer across the CEM and AEM (internal diffusion kinetics) and not the DBLs facing the *D* compartment. The mutual diffusion coefficient of H^+^/Na^+^ ions in the CEM exceeds the corresponding value for the OH^−^/Cl^−^ ions in the AEM (calculated similarly as in [5]), which is expressed in larger values of the fluxes through the corresponding membranes of the former compared to the latter (Figure 4).

Further, in the course of ND, the pH behavior in the *D* compartment follows different trends for each of the considered cases up to approximately 2000 min. Let us consider these differences.

In case 1, as the concentration of the solution in the *D* compartment decreases, the role of the DBLs (where the mutual diffusion coefficient for the OH^−^/Cl^−^ ions is higher than for the H^+^/Na^+^ ions) in controlling the transport kinetics increases and becomes comparable to the role of the membranes. Thus, the ion fluxes through the AEM + adjacent DBLs and CEM + adjacent DBLs are equalized (at *t* ≈ 500 min, Figure 4), which results in an almost constant pH value (Figure 3a). Later, the concentration of the solution decreases to a threshold value [14], after reaching which the process begins to be controlled by external diffusion kinetics since the transfer in the DBLs becomes the limiting stage. The mutual diffusion coefficient of OH^−^/Cl^−^ ions in solution is approximately 20% greater than that of H^+^/Na^+^ ions [5,17], therefore, there is a trend towards an increase in pH.

In case 2, using a lower initial acid concentration leads to a decrease in H^+^ ion fluxes through the CEM as compared to case 1. As a result, at the beginning of the process, the deviation of the pH value from the initial one turned out to be less pronounced (Figure 3a). As a consequence, when the external diffusion kinetics takes over the main role in controlling the process, the increase in pH is more prominent and occurs earlier than in case 1.

In case 3, the initial concentration of alkali was doubled compared to case 1. The ER across the AEM quickly becomes the determining factor of the process, which is expressed in a sharp increase in pH in the *D* compartment even before reaching the threshold concentration at which the external diffusion kinetics begins to dominate. Apparently, this behavior of the system is due to a significant difference in the concentration gradient of OH^−^ ions between the *B* and *D* compartments and the concentration gradient of H^+^ ions between the *A* and *D* compartments.

**Figure 5:**
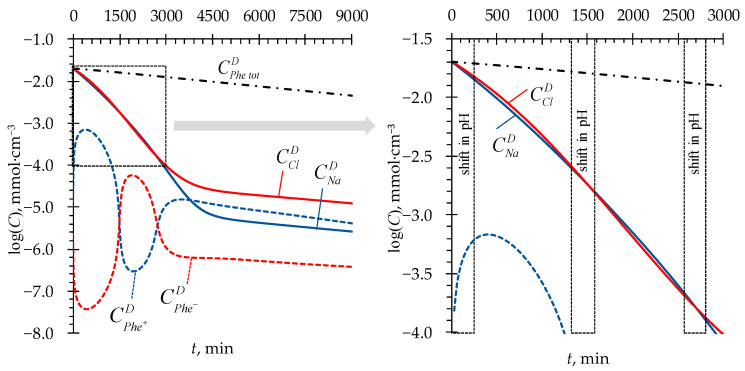
Simulated time dependencies of Na^+^, Cl^−^ (solid lines), Phe^+^, Phe^−^ (dashed lines), and Phe^tot^ (dash-dotted line) concentrations in the *D* compartment. Case 1 is considered (Table 2).

After approximately 2500 min of the process, the model predicts one more shift in pH toward acidic values in all three considered cases. By this time, due to a greater ER across the AEM + adjacent DBLs, the concentration of Cl^−^ ions becomes lower than that of Na^+^ ions (Figure 5). This makes the ER across the CEM + adjacent DBLs greater again and causes the pH in the *D* compartment to decrease (Figure 3a). As a result, after about 3000 min of the process, the concentration of Na^+^ ions becomes lower than that of Cl^−^ ions (Figure 5), and the balance of ER between ion-exchange membranes shifts in favor of the AEM + adjacent DBLs. However, by this time, the concentration of mineral salt ions becomes very low (of the order of the Phe charged forms concentrations), yielding very low values of ion fluxes. Thus, the further changes in pH become very time-expanded. In Figure 3a, starting from approximately 3750 min, the pH time dependence has a shape of a straight line. When the concentration of mineral salt tends to zero, the system tends to an equilibrium state described by Equations (8) and (9), and pH in the *D* compartment tends to the value of pI of Phe. Further, the transport of H^+^ and OH^−^ ions into the *D* compartment will stop since these ions will be spent on local protonation/deprotonation of the zwitterionic form of Phe in membranes, followed by transfer of charged Phe^+^ and Phe^−^ species towards *A* and *B* compartments from the CEM and AEM, respectively. The limiting stage of amino acid transport will be its diffusion through the DBLs to the membrane surfaces facing the *D* compartment.

### 4.2. Phenylalanine Losses

One of the goals of this study is to estimate the Phe losses and to elucidate the mechanisms standing behind the Phe transport in the studied ND system.

The Phe transport is carried out by the diffusion of its charged (Phe^+^, Phe^−^) and zwitterionic (Phe^±^) forms through the membranes and DBLs.

If the solution pH in the *D* compartment deviates from pI of Phe, the protonation/deprotonation of Phe occurs to form its charged forms that are transferred through the membranes as counterions: Phe^+^ through the CEM and Phe^−^ through the AEM. An additional mechanism of Phe transport is so-called facilitated diffusion [24]. It is known from the literature that the pH in the ion-exchange membrane differs from that in the equilibrium solution by 1–2 units [19] (it is lower in the CEM and higher in the AEM due to the Donnan effect [25]). Entering the membrane, the Phe zwitterion becomes protonated (in CEM) or deprotonated (in AEM) to form additional Phe^+^ or Phe^−^ ions, respectively. This provides an increase in the concentration of charged forms of Phe in the corresponding membranes and intensifies the Phe flux. The limiting factor of such a transport mechanism is the Phe^±^ diffusion rate through the DBLs towards the membranes in the *D* compartment. Thus, apparently, the loss of Phe associated with the transport of its zwitterionic form cannot be avoided by controlling the pH of the solution in the desalination compartment.

However, one may try to minimize the loss of Phe transported in the form of charged ions that appeared in the *D* compartment as a result of the change in pH caused by the counter fluxes of H^+^ and OH^−^ ions.

**Figure 6:**
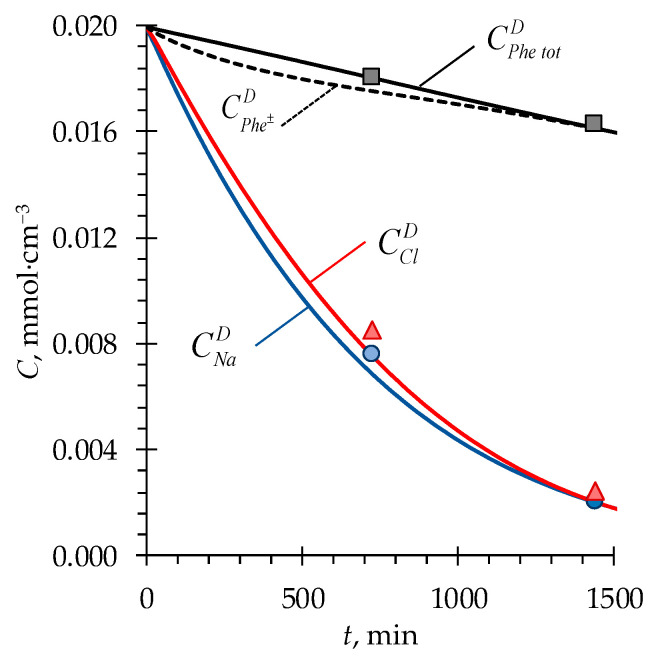
Experimental (markers) and simulated (lines) time dependencies of Na^+^, Cl^−^, Phe^±^, and Phe^tot^ concentrations in the *D* compartment. Case 1 is considered (Table 2).

In the developed model, accounting for the chemical equilibrium of Phe as a function of local pH in solutions and membranes (Equations (8) and (9)) made it possible to describe adequately the experimentally determined concentrations of phenylalanine and mineral salt ions in the solution being desalted (Figure 6).

Knowing the Phe concentrations, the loss of amino acid was calculated by the equation:

(27) $$L=100\left(C_{Phe\;tot,0}^{D}-C_{Phe\;tot,t}^{D}\right)/C_{Phe\;tot,0}^{D}$$

where $C_{Phe\;tot,0}^{D}$ is the initial concentration of Phe in the *D* compartment (at *t* = 0), $C_{Phe\;tot,t}^{D}$ is the sum of the Phe concentrations in all its forms (Phe^+^, Phe^−^, Phe^±^) in the *D* compartment at a given moment in time (*t*).

**Figure 7:**
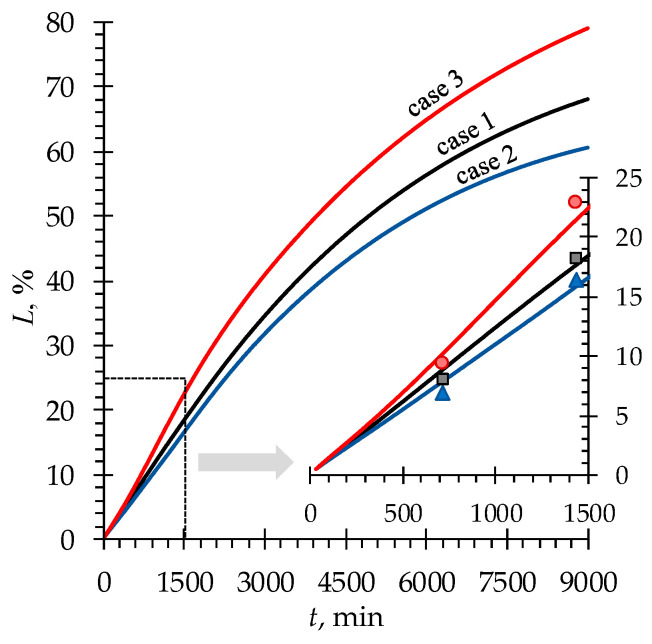
Simulated (lines) and experimentally determined (markers) time dependencies of Phe^tot^ loss in the *D* compartment for the three considered cases (Table 2).

Figure 7 shows that the least loss of Phe is in case 2 (among the three considered cases). In this case, the minimal amount (among all the cases) of Phe^+^ and Phe^−^ ions transferred from the D compartment is conditioned not only by the magnitude of pH deviations from the value of pI but also by the duration of such deviations (Figure 3a).

The question of practical importance is what Phe losses can be expected in the ND of an equimolar NaCl + Phe solution depending on the demineralization rate, calculated by the formula:

(28) $$DR=100\left(C_{NaCl,0}^{D}-C_{NaCl,t}^{D}\right)/C_{NaCl,0}^{D}$$

where $C_{NaCl,0}^{D}$ is the initial concentration of NaCl in the *D* compartment (at *t* = 0), $C_{NaCl,t}^{D}$ is the NaCl concentration in the *D* compartment at a given moment in time (*t*).

**Figure 8:**
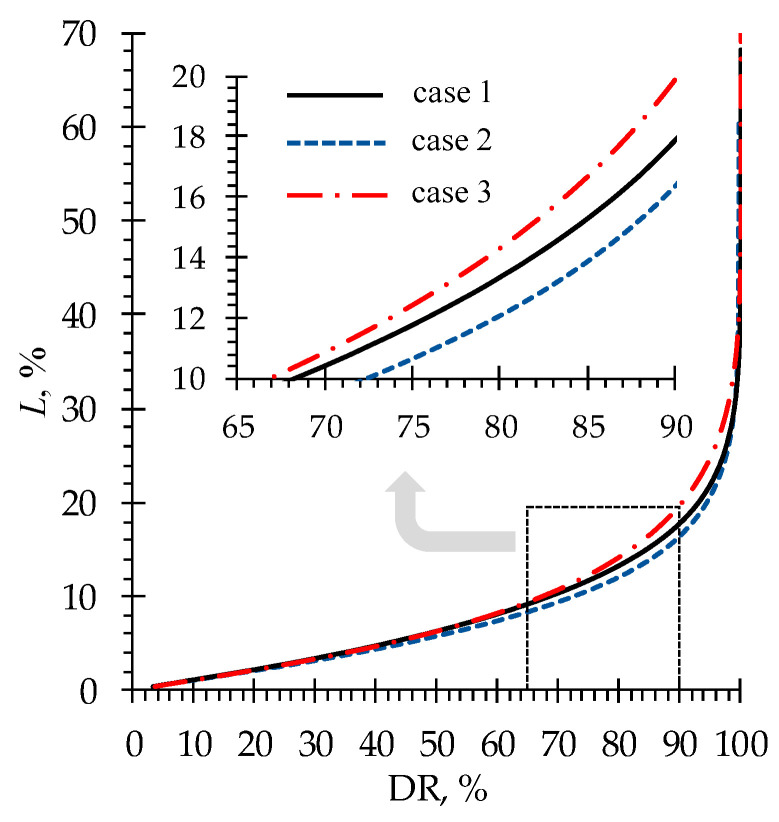
Simulated dependencies of Phe loss as a function of demineralization rate of the solution in the *D* compartment for the three considered cases (Table 2).

In the ND experiment, 90% DR was achieved, accompanied by minimal Phe losses of about 16% (case 2). Modeling predicts a steep increase in phenylalanine losses when the DR is higher than 95% (Figure 8). At such negligibly small concentrations of mineral salt ions, the ER across the membranes is very low. Thus, a lot of time is required to achieve even an insignificant increase in the DR. During this time, the diffusion of Phe^±^ zwitterions leads to an increase in phenylalanine losses. Nevertheless, simulations show that it is possible to achieve a highly demineralized solution characterized by a DR of 99.9% with phenylalanine losses of about 42%.

Thus, this study has shown that ND can be used to effectively demineralize an amino acid solution, and the control of target product losses is possible by selecting process conditions. The developed model allowed a deeper understanding of the mechanisms that determine the behavior of the ND system, which will permit the ability to deliberately set the optimal parameters. Therefore, it will be possible to increase the attractiveness of ND and simplify the task of controlling the pH during the demineralization of amino acid solutions in comparison with other methods. For example, during electrodialysis demineralization of amino acid solutions, reagent-free pH control is possible by controlling the water splitting process (which can occur at interface boundaries in over-limiting current modes [26]). This is possible by regulating the applied electric current density [27] or by selecting different types of membranes, for example, bipolar ones [28]. However, the problem of water splitting control is rather sophisticated, and this approach requires the application of high voltages [29], thereby, adding chemical reagents is still a common practice used to adjust solution pH in electrodialysis [30]. As for diffusion dialysis, which is also widely used for the demineralization of ampholyte-containing solutions, the control of the pH is possible only with the use of additional reagents. Moreover, the values of fluxes that can be achieved in diffusion dialysis are very low [31], thus, a great number of membranes are required for the demineralization of solutions [32]. The advantage of ND is the relatively high rate of solution desalination. Due to the neutralization reaction in the *D* compartment, a high concentration gradient of H^+^ and OH^−^ ions is maintained, resulting in a relatively high magnitude of ion fluxes through membranes (comparable to ion fluxes in electrodialysis, ≈10^−5^ mmol·cm^−2^·s^−1^ [29,33]). This allows for achieving relatively high performance with relatively small plant sizes.

## 5. Conclusions

A non-steady state model that describes the neutralization dialysis demineralization of a mixed sodium chloride and phenylalanine solution was proposed. The model takes into account the characteristics of membranes that are important for practice (thickness, ion-exchange capacity, conductivity). In contrast to the previously developed models, the new one considers the local equilibrium of phenylalanine protolysis reactions in solutions and membranes and the phenylalanine transport in its charged and zwitterionic forms through membranes.

A series of experiments on neutralization dialysis demineralization of the NaCl and phenylalanine mixed solution was carried out. In order to minimize phenylalanine losses, the solution pH in the desalination compartment was controlled by changing the concentrations of the solutions in the acid and alkali compartments of the dialysis cell.

The validity of the model was verified by comparison of simulated and experimental time dependencies of solution electrical conductivity and pH, as well as the concentration of mineral salt ions and phenylalanine in the desalination compartment of the neutralization dialysis cell.

The results of simulations show that the phenylalanine losses associated with the facilitated diffusion transport mechanism can not be avoided in neutralization dialysis due to the H^+^ and OH^−^ ions fluxes directed oppositely to the fluxes of phenylalanine zwitterions. However, it is possible to lower the transport (consequently, the losses) of charged forms of phenylalanine that formed in the desalination compartment due to pH changes. This reduction can be achieved by regulating the pH in the desalination compartment in a way to keep it as close as possible to the value of amino acid pI. In this study, pH was regulated by influencing the fluxes of H^+^ and OH^−^ ions through the membranes by changing the solution concentrations in the acid and alkali compartments of the cell.

In the experiments carried out, the demineralization rate has reached 90%, accompanied by the minimal phenylalanine losses of about 16%. In the studied system, this result was achieved by using the acid concentration half as much as the alkali concentration. Modeling predicts a steep increase in phenylalanine losses when the demineralization rate is higher than 95%. Nevertheless, simulations show that it is possible to achieve a highly demineralized solution (by 99.9%) with phenylalanine losses amounting to 42%.

## Figures and Tables

**Table 1:** Some physicochemical characteristics of CSE and ASE membranes. Data taken from [11].

Membrane	CSE	ASE
Ion-exchange capacity, mmol·cm^−3^	1.85	1.99
Water content, %	42.0	24.4
Conductivity (in 0.1 M NaCl), mS·cm^−1^	7.37	3.77
Conductivity (in 0.1 M Phe, CSE at pH = 0.5, ASE at pH = 13.1), mS·cm^−1^	5.67	2.32
Conductivity (CSE in 0.1 M HCl, ASE in 0.1 M NaOH), mS·cm^−1^	71.12	6.76
Thickness in swollen state, microns	140	150

**Table 2:** Initial concentrations in ND system compartments.

Case	$C_{HCl}^{A}$, mol·L^−1^	$C_{NaOH}^{B}$, mol·L^−1^	$C_{NaCl}^{D}$, mol·L^−1^	$C_{Phe^{tot}}^{D}$, mol·L^−1^
case 1	0.1	0.1	0.02	0.02
case 2	0.05	0.1		
case 3	0.1	0.2		

## Data Availability

Not applicable.

## Appendix A

**Table A1:** Parameters of the model.

Parameter	Value	Units	Description
$d^{c}$ and $d^{a}$	140 and 150	microns	CEM and AEM thickness, respectively *
$\delta^{k}$	85		The thickness of diffusion boundary layers **, *k* = *I*, *II*, *III*, *IV* (Figure 1)
$C_{NaCl}^{D}$	0.02	mol·L^−1^	Initial molar concentration of salt in the desalination compartment *D* (Table 2)
$C_{HCl}^{A}$	0.1 or 0.05		Initial molar concentration of acid in the acid compartment *A* (Table 2)
$C_{NaOH}^{B}$	0.1 or 0.2		Initial molar concentration of alkali in the alkali compartment *B* (Table 2)
$C_{Phe^{tot}}^{D}$	0.02		Initial molar concentration of phenylalanine in the desalination compartment *D* (Table 2)
*V^A^* and *V^B^*	2	L	Volume of solutions in the acid and alkali compartments, respectively
*V^D^*	0.5		Volume of the solution in the desalination compartment
$Q^{c}$ and $Q^{a}$	1.85 and 1.99	mmol·cm^−3^	Ion-exchange capacity of CEM and AEM, respectively (in the swollen state) *
$D_{Na}$	1.34·10^−5^	cm^2^·s^−1^	Diffusion coefficients of Na^+^, Cl^−^, H^+^, OH^−^, Phe^+^, Phe^−^, and Phe^±^ ions in solution at infinite dilution [22,34]
$D_{Cl}$	2.03·10^−5^		
$D_{H}$	9.31·10^−5^		
$D_{OH}$	5.26·10^−5^		
$D_{Phe^{+}}$	6.7·10^−7^		
$D_{Phe^{-}}$	6.7·10^−7^		
$D_{Phe^{\pm }}$	6.7·10^−7^		
${\bar{D}}_{Na}^{c}$	3.25·10^−7^		Diffusion coefficients of Na^+^, H^+^, Phe^+^ ions in CEM and Cl_−_, OH^−^, Phe^−^ ions in AEM **
${\bar{D}}_{H}^{c}$	9.0·10^−6^		
${\bar{D}}_{Phe^{+}}^{c}$	1.1·10^−7^		
${\bar{D}}_{Cl}^{a}$	2.25·10^−7^		
${\bar{D}}_{OH}^{a}$	7.8·10^−7^		
${\bar{D}}_{Phe^{-}}^{a}$	0.75·10^−7^		
${\bar{D}}_{Phe^{\pm }}^{c}$ and ${\bar{D}}_{Phe^{\pm }}^{a}$	3.3·10^−8^ and 2.3·10^−8^		Diffusion coefficients of Phe^±^ ions in CEM and AEM, respectively **
$K_{w}$	10^−14^	mol^2^·L^−2^	Water ionization constant
$K_{1}$	6.31·10^−3^	mol·L^−1^	Equilibrium constant for chemical reaction (6) [22]
$K_{2}$	4.90·10^−10^		Equilibrium constant for chemical reaction (7) [22]
$K_{H,Na}^{c}$ and $K_{H,Phe^{+}}^{c}$	1	–	Nikolskii equilibrium constant for exchange between H_+_ and Na^+^, H^+^, and Phe^+^ in CEM
$K_{OH,Cl}^{a}$ and $K_{OH,Phe^{-}}^{a}$	1	–	Nikolskii equilibrium constant for exchange between OH^−^ and Cl^−^, OH^−^ and Phe^−^ in AEM
*T*	298	K	Absolute temperature
*R*	8.314	J·(mol·K)^−1^	Gas constant
*F*	96,485	C·mol^−1^	Faraday constant

* Parameters obtained from independent experiments (Table 1, data taken from [11]). ** Fitting parameters of the model.

**Table A2:** List of abbreviations and superscripts/subscripts.

Abbreviations	Description
AEM	anion-exchange membrane
CEM	cation-exchange membrane
DBL	diffusion boundary layer
DR	desalination rate
ER	exchange rate
ND	neutralization dialysis
Phe	phenylalanine
**Superscripts/subscripts**	
Superscripts “*c*” and “*a*”	relates to CEM and AEM, respectively
Superscripts “*A*”, “*B*”, and “*D*”	relates to acid, alkali (base), and desalination compartments, respectively
Subscript “*j*”	relates to a certain species. The type of species described by the index *j* is indicated in the place of its appearance
